# 

**Published:** 1869-02

**Authors:** 


THE
### IOWA MEDICAL JOURNAL

A BI-MONTHLY OF 32 PAGES.

*Price, Two Dollars per Annum, Payable in Advance.*

GENTLEMEN OF THE PROFESSION:

We regret the irregularity that has thus far characterized the issue of the Fifth Volume of THE JOURNAL. But when we say that sickness of the Editor, and a want of proper support from the profession, has caused it, we say but the truth. We again appeal to our friends to give us the proper encouragement, and we will try again; but if the profession expect us to edit it, pay for it, and take the curses which attach to the position, they will find an editor suddenly withdraw from the assumed honors, fully convinced that *glory* of this kind will not pay.

